# A decision analysis for periapical surgery: Retrospective Study

**DOI:** 10.4317/jced.53334

**Published:** 2018-09-01

**Authors:** Göksel Şimşek-Kaya, Nesrin Saruhan, Günay Yapıcı-Yavuz, Ümit Ertaş

**Affiliations:** 1 Department of Oral and Maxillofacial Surgery, Faculty of Dentistry, Akdeniz University, Antalya, Turkey; 2 Department of Oral and Maxillofacial Surgery, Faculty of Dentistry, Osmangazi University, Eskişehir, Turkey; 3 Department of Oral and Maxillofacial Surgery, Faculty of Dentistry, Adıyaman University, Adıyaman, Turkey; 4 Department of Oral and Maxillofacial Surgery, Faculty of Dentistry, Atatürk University, Erzurum, Turkey

## Abstract

**Background:**

Periapical surgery is now a reliable therapeutic procedure for the treatment of teeth with periapical lesions, particularly when orthograde retreatment is problematic. However, little information is available regarding treatment planning of cases referred for periapical surgery. Therefore, this study was conducted to analyze and evaluate the factors that affect the decision-making process for periapical surgery.

**Material and Methods:**

This study retrospectively assessed clinical and radiographic data from patients undergoing periapical surgery. The factors involved in deciding to perform periapical surgery were classified into technical, biological, and combined factors.

**Results:**

Out of 821 patients, 544 (66.3%) underwent endodontic treatment/retreatment, 204 (24.8%) were treated with coronal restorations and 60 (7.3%) were treated with post. Periapical surgery was indicated for biological reasons in 35% of patients and for technical reasons in 17.9%. The common biological factor was persistent clinical symptoms (19.7%). The most common technical cause was failure of previous endodontic treatment (66.3%). Nearly half of all periapical lesions (45%) were <5 mm in size. Periapical surgery was justified in only 434 (52.9%) subjects.

**Conclusions:**

We suggest that it is very important for patients to be informed and encouraged about endodontic retreatment in order to reduce unnecessary surgical procedures.

** Key words:**Periapical surgery, case selection, treatment planning.

## Introduction

Persistent apical periodontitis following orthograde root-canal treatment is common among adult populations in various countries, with prevalence rates varying between 27%-70% and increasing with age (1). Conventional root-canal treatment is considered to be the best method of managing periapical disease, with success rates varying between 48%-98% (2). If root canal treatment fails, the reasons for this must be accurately assessed before any further intervention. Whenever possible, nonsurgical retreatment is regarded as the treatment of choice. However, where nonsurgical retreatment is not an option, periapical surgery (endodontic surgery) is considered to be a viable alternative (2). In order to eliminate existing extraradicular infections, foreign bodies and cystic tissue, periapical tissue is debrided by complete curettage in periapical surgery (3,4). In fact, Kim and Kratchman (5) suggested that surgical treatment can be more conservative than non-surgical treatment in certain cases, particularly in the frequently observed instance of a tooth with satisfactory endodontics, a new post-and-coronal restoration, but a refractory or growing periapical lesion. Breaking or disassembling the coronal before removing the post and then retreating the canal, the authors argue, would be more traumatic, time-consuming and expensive and the results more uncertain than a root-end microsurgical approach (5).

The decision to perform periapical surgery should be based on comprehensive examination of the patient’s dental, oral and medical conditions. In fact, however, treatment decisions are often based on the preferences and experience of the clinician. Moreover, patients often tend to choose the least costly option, i.e. tooth extraction, overlooking the functional, esthetic and psychological results of tooth loss (6). Few previous studies have assessed the relative importance of the different factors involved in the decision to perform periapical surgery (7). Despite the fact that case and treatment selection represent the first stage of treatment, only three retrospective studies to date have investigated the decision-making process involved in periapical surgery (7-9), which has been examined mainly in terms of contemporary microsurgical techniques and prognostic factors (10,11). Therefore, this study was conducted to analyze and evaluate the factors that affect the decision-making process for periapical surgery.

## Material and Methods

-Study population

This retrospective study was based on the patients treated with apical surgery between January 2000 and December 2012 at the Oral and Maxillo-Facial Surgery Department of Atatürk University in Erzurum, Turkey. Records included referral letters and existing radiographs (panoramic, periapical) as well as initial clinical examinations, principal symptoms, history of the referred tooth, summary of the treatment provided before referral, and general medical status. The criteria for inclusion in the study were good quality radiographs, complete data in the dental charts (age, gender, tooth type, information whether the previous root canal treatment was primary or retreatment, information whether the previous periapical surgical treatment was primary or retreatment, coronal restorations, and clinical symptoms). Since these radiographs had originally been taken for definite radiological diagnosis previously and the analysis had an observational structure, it was therefore not necessary to seek ethical approval for this study. Patients who were missing records or had poor quality radiographs were excluded from the study.

-Study design

Patient records were examined simultaneously by 4 oral and maxillofacial surgeons. Physical status of patients was classified according to the American Society of Anesthesiology (12). Radiographs were assessed by visual examination using an x4 magnifying lens and a transparent, flexible ruler (mm). The factors involved in deciding to perform periapical surgery in this study were classified into technical, biological, and combined factors. The techical factors were root-canal treatment, post, coronal restoration, broken instrument, extruded material, calcification, and others. The biological factors included persisting clinical symptoms, periradicular lesions such as cyst. In cases where technical and biological factors were occuring together and were both involved in the decision making process they were considered to be combined. Then, the following parameters were assessed and recorded as either adequate or inadequate based on the following criteria.

a) Coronal restoration 

Marginal adaptation of the restoration was considered adequate in the absence of any evidence of radiolucent images between the restoration margin and the remaining ccoronal restoration (8,13,14);

b) Endodontic treatment 

Treatment was considered adequate when the canal filling was radiographically dense and homogenous and extended to within at least 1 mm of the anatomic apex (8,15,16), root canals did not appear underprepared in width, and no voids were observed between the canal filling and walls (8);

c) Posts

Posts were identified by comparing a patient’s clinical records and radiographic findings of an area of increased radiopacity in the middle and cervical thirds of the root canal compatible with the post description in the file (17). Posts were regarded as sufficient if located along the axis of the root canal with no radiographic voids in the filling material between the post and the canal walls. Roots with posts were divided into two groups according to length of the post (longer or shorter than 5 mm, as measured on a radiograph), and posts exceeding 5 mm were regarded as potentially unsuitable for removal or drilling (8);

d) Periapical lesions

A periapical lesion was defined as any radiolucent image exceeding 1 mm (15,16). Both the smallest and largest diameters of each lesion were measured, and mean diameters were calculated. Lesions with a mean diameter of > 5 mm were classified as large lesions, and those with lesions of ≤5 mm were classified as small lesions (18).

The clinical status of the referred tooth and its periapical tissue was recorded and assessed. For teeth judged to have inadequate root-canal fillings, surgeons evaluated the potential complexity involved in nonsurgical retreatment and classified teeth as follows.

1) Teeth that could be retreated following the successful removal of the coronal restoration; and 

2) Teeth that required surgery because retreatment would be impossible or a high risk due to blocked canals, broken instruments, calcification, etc.

Statistical analysis was performed using the Statistical Package for the Social Sciences software version 21.0 (SPSS Inc., Chicago, IL, USA). For the test on the relevant significance of the frequencies, descriptive statistics were used.

## Results

**Figure 1:**
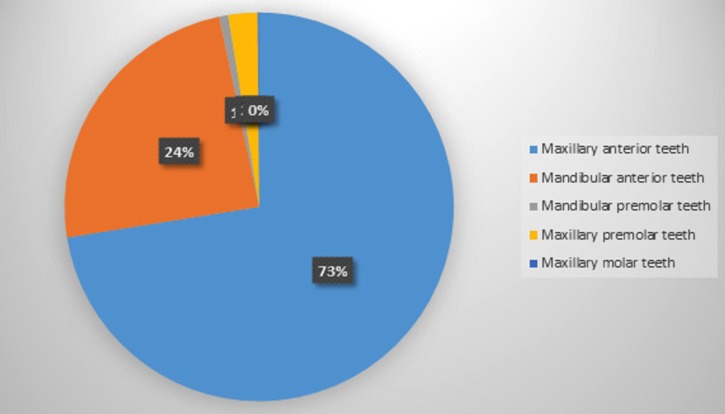
Distruption of the number of treated teeth in the two arches by tooth type.

**Figure 2:**
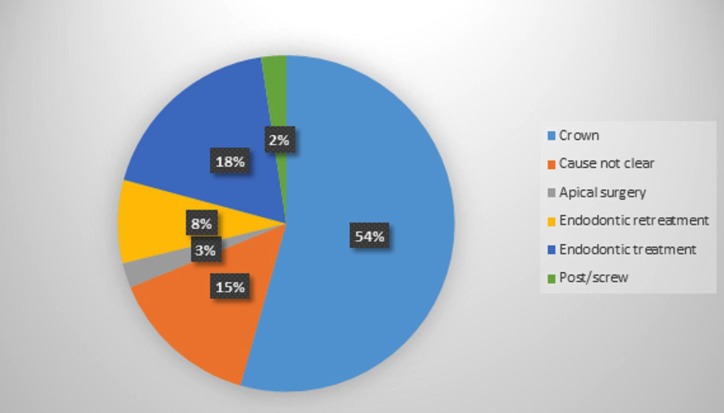
Relative proportions of probable cause of cyst.

**Figure 3:**
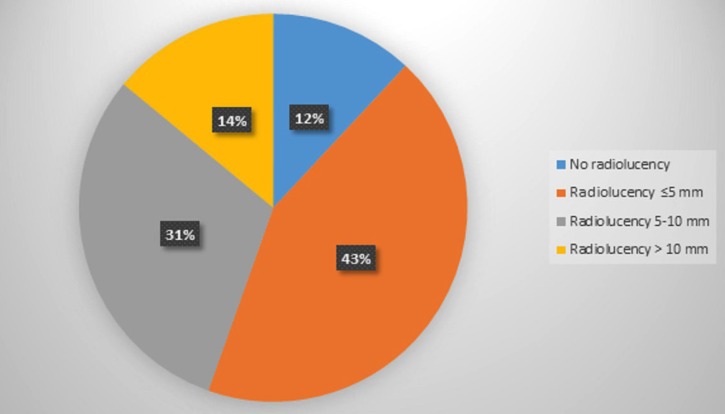
Distruption of the treated teeth according to periapical radiolucency status.

Seven patient’s records (13 teeth) were excluded from the study because there were no preoperative radiographs, and one of them had no treatment record sheets. Therefore, while 828 patient’ records were initially considered, 821 eventually met the enrollment criteria. Of the 821 patients (1,110 teeth) in the study (471 female, 350 male; age range: 12-75 years; mean age: 26.6) 68% were individuals referred by general dental practitioners, and the remaining 32% were referred by the Atatürk University Dental Faculty’s Department of Endodontics. Patient anamneses showed all patients to have Class I or Class II physical status according to American Society of Anesthesiology (ASA) criteria (19). The most frequently assessed teeth were maxillary anterior teeth (Fig. 1). Apical surgery was indicated for biological reasons in 35% of patients and for technical reasons in 17.9%. The common biological factor was persistent clinical symptoms (19.7%) in this study. The other biological factor was cyst (15.2%). The most common technical cause was failure of previous endodontic treatment/retreatment (66.3%) ( Table 1- Table 3). 79.9 % of coronal restorations were adequate and 71.7 % of posts were longer than 5 mm. Coronal restoration was found to be the most common probable factor in cyst formation (Fig. 2). These cysts were identified histopathologically as radicular cysts. Lesions were associated with 1 tooth in 432 patients, 2 teeth in 197 patients, 3 teeth in 58 patients, 4 teeth in 23 patients, 5 teeth in 1 patient, 6 teeth in 1 patient and 7 teeth in 1 patient. Less than half (45%) of all apical lesions exceeded 5 mm in size (Fig. 3).

**Table 1:**
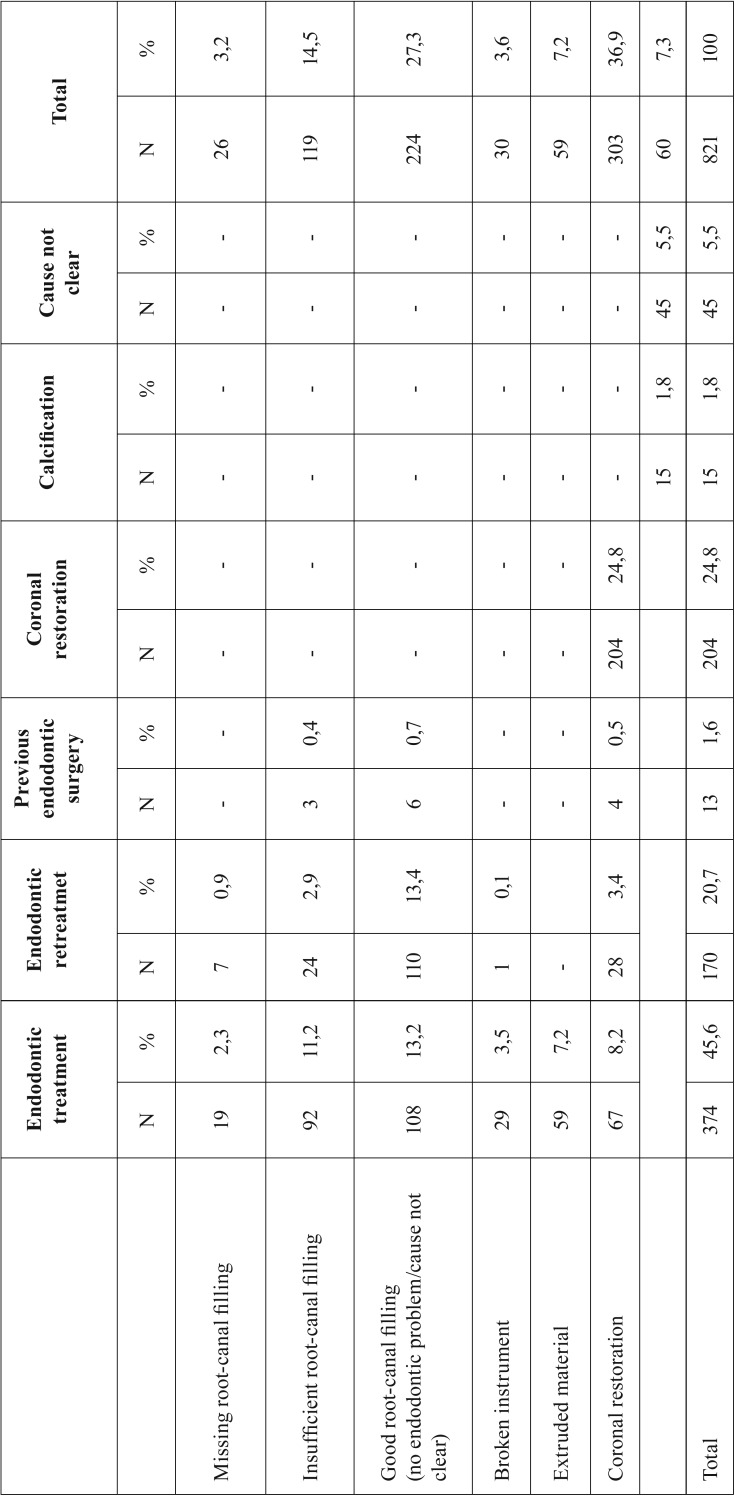
Probable technical causes in patients undergoing apical surgery.

**Table 2:**
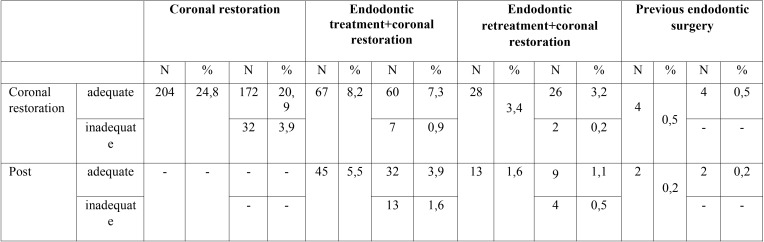
Distribution of patients undergoing coronal restoration and post.

**Table 3:**
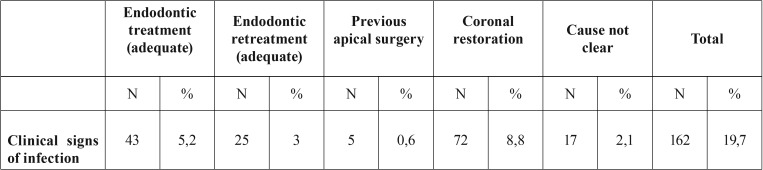
Distribution of persistent clinical symptoms.

## Discussion

In deciding whether or not to perform endodontic surgery, clinicians need to weigh a number of factors, including whether or not a patient’s symptoms include discomfort; whether the goal of treatment is esthetic and/or functional improvement; whether or not surgery has been performed previously, and if so, the outcome; whether or not a patient has a medical history that might influence treatment; clinical and radiological findings; experience of the clinician; and the economic status of the patient (19). Other patient-related factors that play a role in the choice between endodontic retreatment and surgical intervention include the risk of complications due to proximity to nerves and other structures and the presence of prosthetic restorations (8,19). It is likely that many cases considered suitable for retreatment were referred for surgery not as a result of acute pain and swelling, but because the dentist considered retreatment to be laborious and time-consuming (20). Whether this constitutes a valid reason is questionable. In this regard, the present study as well as previous studies (7-9) are limited by a lack of data regarding patient socioeconomic status.

El-Swiah and Walker (7) stated that indications for periapical surgery depended on biological and technical factors, with biological factors involved in 60% of decisions to perform apical surgery and technical factors involved in the remaining 40%. The most common biological factors were the persistence of clinical symptoms after conventional root canal treatment (54.1%) and the persistence of a periradicular lesion (44%), whereas the most common technical factors were the presence of a post and coronal restoration (60.4%) and the presence of a coronal restoration without a post (31%). Another retrospective study by Abramovitz *et al.* (8) found 70% of teeth were indicated for periapical surgery due to technical factors, with 40% involving coronal restorations with posts and 30% involving coronal restorations without posts (8), while a retrospective study by Beckett (9) found 50% of periapical surgery patients had teeth with post/screw. In the present study, persisting clinical symptoms was the most common biological factor (19.7%) and failure of previous endodontic treatment was the most common technical factor (66.3%); in addition, in 30.6% of patients, both biological and technical factors were involved in the decision to perform periapical surgery, and which factor was more important could not be determined ( Table 3, Fig. 2).

Beckett (9) posited that short posts and/or a radiographically detectable gap between post and root canal were not a technical obstacle to periapical surgery. Abramovitz *et al.* (8) also suggested that coronal restorations with no posts, or with posts shorter than 5 mm, should not be regarded as an insuperable technical impediment to endodontic treatment. Thus, while they found that teeth with post or coronal restoration represented approximately 75% of cases referred for periapical surgery for technical reasons, in view of the possibility of treating some teeth with post/coronal restoration endodontically, the authors considered surgery to be necessary in only 14% of the referred teeth (8). In the present study, 28.3% of posts were either shorter than 5 mm or were loose and could safely be removed; in 26.7% of cases, there was no root-canal filling or the filling was of poor quality; and the presence of a coronal restoration represented a technical obstacle in 24.8% of patients. It is possible that the decision to perform periapical surgery may have been influenced by the possibility of fracture during restoration extraction leading to sizeable financial costs and prolonging the therapeutic process.

As in previous retrospective studies (6,7), the current study found most of the teeth treated by periapical surgery were maxillary incisors (Fig. 1). This is understandable, since maxillary teeth appear to undergo conventional root canal treatment more often than mandibular teeth (7). In addition, the central incisors were the most common mandibular teeth to undergo periapical surgery (Fig. 1). Given that an uncleaned second canal may be responsible for failure of conventional root canal treatment in mandibular incisors (7), this finding highlights the need for clinicians to investigate the condition of both canals when performing conventional treatment of mandibular incisors.

Significant radiolucency per se does not constitute a contraindication for periapical surgery (6). Although there is insufficient scientific data to either support or reject a “size-based” attitude towards treatment of periapical disease (21), and a number of studies have reported surgical success rates to be lower in teeth with larger lesions compared to teeth with lesions smaller than 5 mm (10,22). Arx *et al.* (6) suggested that with lesions exceeding 10 mm in size, the decision on whether or not extraction should be performed should take into consideration periodontal conditions, such as increased tooth mobility, pain, and other clinical signs and symptoms. Based on these considerations, the authors elected to extract 51.8% of teeth with lesions exceeding 10 mm in their study. In the current study, lesions exceeding 10 mm in size represented only 14% of teeth treated by periapical surgery. Increases in lesion size may be accompanied by an increase in lesion-associated teeth; however, so long as extraction is not required for periodontal reasons, the option of allowing teeth to remain in the mouth should be considered. Based on the facts that biological factors were involved in 35% of patients in this study and that lesion size was >5 mm in 45% of patients, the authors believe, as suggested by El-Swiah and Walker (7), that 5 mm is the size at which a periapical lesion is either identified radiographically or becomes symptomatic and noticeable by the patient.

In a study by Abramovitz *et al.* (8), the authors re-examined patients presenting for apical surgery and concluded that surgery was justified in only 45% of cases. According to Beckett (9) this rate was 65%, while in this study we found 52.9%. Kvist *et al.* (21) reported that only 6% of general dental practitioners considered retreatment in cases of endodontic failure, and another survey reported that the majority of general practitioners preferred surgery as the first option for treating failed endodontic cases; moreover, referral of difficult cases to an endodontist was not common practice (23). A previous study has already highlighted the need for improving the quality of endodontic treatment in Turkey (24), as the authors judged 58.9% of root-canal fillings to be inadequate and found apical periodontitis in 15.8% of root-filled teeth. On the basis of these findings, it may be suggested that the need for apical surgery may be significantly reduced if endodontic treatment is performed using the most current techniques and materials, and if complicated cases, in particular, are referred to an endodontist for treatment.

## Conclusions

In a significant proportion of patients, teeth that underwent periapical surgery, endodontic treatment had been performed at least once previously; however, in spite of this treatment, clinical symptoms persisted, and endodontic surgery was preferred over further endodontic treatment. The choice of treatment may depend on the preference of the clinician as well as on the poor experience previously experienced by the patient during dental treatment. Our data also revealed that periapical surgery was justified in 52.9% of the cases. We suggest that it is very important for patients to be informed and encouraged about endodontic retreatment in order to reduce unnecessary surgical procedures.
